# Chromatin Mediation of a Transcriptional Memory Effect in Yeast

**DOI:** 10.1534/g3.115.017418

**Published:** 2015-03-05

**Authors:** Emily Paul, Itay Tirosh, William Lai, Michael J. Buck, Michael J. Palumbo, Randall H. Morse

**Affiliations:** * Laboratory of Molecular Genetics, Wadsworth Center, New York State Department of Health, Albany, New York; † Department of Biomedical Science, University at Albany School of Public Health, Albany, New York; ‡ Department of Molecular Genetics, Weizmann Institute of Science, Rehovot, Israel; § Department of Biochemistry and the Center of Excellence in Bioinformatics and Life Sciences, State University of New York at Buffalo, Buffalo, New York

**Keywords:** transcription, yeast, Abf1, chromatin, nucleosome, ChIP-seq

## Abstract

Previous studies have described a transcriptional “memory effect,” whereby transcript levels of many Abf1-regulated genes in the budding yeast *Saccharomyces cerevisiae* are undiminished even after Abf1 has dissociated from its regulatory sites. Here we provide additional support for this effect and investigate its molecular basis. We show that the effect is observed in a distinct *abf1 ts* mutant from that used in earlier studies, demonstrating that it is robust, and use chromatin immunoprecipitation to show that Abf1 association is decreased similarly from memory effect and transcriptionally responsive genes at the restrictive temperature. We also demonstrate that the association of TATA-binding protein and Pol II decreases after the loss of Abf1 binding for transcriptionally responsive genes but not for memory effect genes. Examination of genome-wide nucleosome occupancy data reveals that although transcriptionally responsive genes exhibit increased nucleosome occupancy in *abf1 ts* yeast, the promoter regions of memory effect targets show no change in *abf1 ts* mutants, maintaining an open chromatin conformation even after Abf1 eviction. This contrasting behavior reflects different inherent propensity for nucleosome formation between the two classes, driven by the presence of A/T-rich sequences upstream of the Abf1 site in memory effect gene promoters. These sequence-based differences show conservation in closely related fungi and also correlate with different gene expression noise, suggesting a physiological basis for greater access to “memory effect” promoter regions. Thus, our results establish a conserved mechanism underlying a transcriptional memory effect whereby sequences surrounding Abf1 binding sequences affect local nucleosome occupancy following loss of Abf1 binding. Furthermore, these findings demonstrate that sequence-based differences in the propensity for nucleosome occupancy can influence the transcriptional response of genes to an altered regulatory signal.

Abf1 is an essential, abundant general regulatory factor found in *Saccharomyces cerevisiae* and conserved in other fungi (Wapinski *et al.* 2007). Abf1 is involved in transcriptional activation, DNA replication, DNA repair, and gene silencing (Hardy *et al.* 1992; Miyake *et al.* 2002, 2004; Rhode *et al.* 1992). Abf1 regulates approximately 200−300 targets in yeast, including the *ABF1* gene itself, which it negatively regulates (Miyake *et al.* 2004; Yarragudi *et al.* 2007). Regulatory responsibilities include transcriptional activation/repression of various gene families involved in amino acid transport, carbon source regulation, sporulation, meiosis, and mitochondrial function (de Boer *et al.* 2000; de Winde and Grivell 1992; Loo *et al.* 1995; Schlecht *et al.* 2008; Silve *et al.* 1992). Previous work demonstrated that Abf1 is able to outcompete histones for occupancy of its binding site at natural and synthetic locations (Venditti *et al.* 1994; Yarragudi *et al.* 2004). Additionally, increased nucleosome occupancy was observed at numerous promoter sites throughout the yeast genome upon Abf1 depletion, indicating that Abf1 maintains an open chromatin conformation at its binding sites (Badis *et al.* 2008; Ganapathi *et al.* 2011; Hartley and Madhani 2009).

Experiments performed previously in *abf1-1 ts* yeast demonstrated a transcriptional memory effect occurred at select Abf1-regulated targets at the restrictive temperature (Schroeder and Weil 1998). Continuous binding of transcriptional activators to their cognate promoter binding sites is generally viewed as being necessary for ongoing transcription (Ho *et al.* 1996). However, Schroeder and Weil (1998) showed that in an *abf1-1 ts* mutant, mRNA levels of several genes known to depend on Abf1 binding sites for activation were undiminished after 1 hr at the restrictive temperature (Schroeder and Weil 1998). DMS footprinting showed loss of Abf1 binding under these same conditions, suggesting that these target genes retained a molecular memory of Abf1 binding that allowed their continued transcription at the restrictive temperature. Later examination of genome-wide expression in *abf1-1 ts* yeast revealed at least 80 Abf1 probable regulatory targets that did not show significantly reduced transcription at 37°, whereas 37 stringently defined Abf1-regulated target genes showed reduced mRNA levels by at least 1.5-fold (Yarragudi *et al.* 2007). We refer to these two classes of Abf1 targets, which in fact represent two ends of a continuum, as “memory effect” and “transcriptionally responsive” genes, respectively.

The molecular mechanisms responsible for the transcriptional memory effect exerted at Abf1-regulated genes are unknown. We examined previously the possibility that memory effect genes use different Abf1 binding motifs than do promoters of transcriptionally responsive targets but found identical Abf1 consensus motifs for the two classes (Yarragudi *et al.* 2007). Here we examine other possible causes of this memory effect and identify an evolutionarily conserved, sequence-directed propensity for nucleosome formation as a distinguishing feature and likely contributing factor to this phenomenon.

## Materials and Methods

### Yeast strains and growth

Yeast strains are listed in Table 1. Binding site mutants were constructed using the *delitto perfetto* technique to replace the 14-bp Abf1 binding site with the sequence CTACTAGTTA or its complement depending on the orientation of the Abf1 binding site (Storici and Resnick 2006). Yeast cells were grown in rich medium, Yeast Peptone Dextrose [1% bacto-yeast extract (10 g/L), 2% bacto-peptone extract (20g/L), 2% glucose (20g/L) in dH_2_O]. For temperature shift experiments, cultures were grown to mid-log phase (OD_600_ = 0.6−1.2), diluted to OD_600_= 0.3−0.5 and allowed to grow for 2 hr at the permissive temperature (25°). Culture density was measured to ensure doubling, and cultures were shifted to the restrictive temperature (37°) by adding prewarmed media and incubated at 37° for 60 min. All other cultures were grown to mid-log phase (OD_600_ = 0.6-1.2), diluted to OD_600_= 0.3-0.5 and allowed to grow for 2 hr at 30°.

**Table 1:** Yeast strains used in this study

Strain	Genotype	Reference
TMY86	*Mat****a*** *ade2-1 his3-11,15 leu2-3,112 trp1-1 ura3-1 can1-100 abf1Δ*::*HIS3MX6 [pRS416-ABF1]*	(Miyake *et al.* 2002)
*abf1-1*	*TMY86-1/pTM629*; *pRS415-abf1-1*	(Miyake *et al.* 2002)
BY4741		Yeast Deletion Library
CBY11502	*Mat a*, *his3Δ1*, *leu2Δ0*, *ura3Δ0*, *met15Δ0*, *abf1-101 ts*::*KanMX*	Gift from C. Boone
Z579	*Mat a*, *his3∆200*, *leu2-3,122*, *ura3-52*, *srb4∆2*::*HIS3 [pCT181/RY2882(SRB4 LEU2 CEN)]*	(Thompson and Young 1995)
Z111	*Mat alpha*, *ura3-52*, *his3∆200*, *leu2-3,112*, *rpb1-1*, *ade2*	(Nonet *et al.* 1987)
EPY10	BY4741/mutant Abf1 binding site (10mer) @ p*RPS28A*	This study
EPY26	BY4741/ mutant Abf1 binding site (10mer) @ p*RPL3*	This study
EPY66	BY4741/ mutant Abf1 binding site (10mer) @ p*CNB1*	This study
EPY67	BY4741/ mutant Abf1 binding site (10mer) @ p*ARO3*	This study

### Chromatin immunoprecipitation (ChIP) and library preparation and amplification

Whole-cell extracts were prepared from 50 mL of yeast cells as described previously (Ansari *et al.* 2012). Conventional ChIP was performed as described previously, using 180 μL of whole-cell extracts with the following antibodies: αAbf1 (5 μg; Santa Cruz Biotechnology; Santa Cruz, CA), αTBP (2.5 μg; gift from PA Weil, Vanderbilt University, TN), and αRpb3 (1 μg; Neoclone; Madison, WI; www.neoclone.com). Bar-coded libraries for ChIP-seq were prepared as recently described (Paul *et al.* 2015) and were sequenced at the University of Buffalo Next-Generation Sequencing and Expression Analysis Core (University at Buffalo, State University of New York; Buffalo, New York).

### RNA isolation and cDNA synthesis

Total RNA isolation was carried out from 10-mL cultures using the Hot Phenol protocol adapted from Schmitt *et al.* (1990). RNA levels were quantified by quantitative real-time polymerase chain reaction of cDNA generated using First-Strand cDNA Synthesis Kit for Real-Time PCR (USB/Affymetrix, Cleveland, OH). For cDNA synthesis, either a mix of oligo dT and random hexamers (proprietary concentrations) or a mix of appropriate gene-specific oligonucleotides was used for reverse transcriptase primers. Reactions were run at 44° for 1 hr and 92° for 10 min, as per manufacturer’s recommendation.

### Quantitative Real-Time Polymerase Chain Reaction

Quantitative real-time polymerase chain reaction was conducted as described previously (Ansari *et al.* 2012) using a StepOnePlus Real Time PCR System (Applied Biosystems/Life Technologies, Green Island, NY). Technical replicates of individual 12.5-μL reactions were averaged prior to averaging biological replicates and calculating standard deviations. Reactions were run on Fast protocol (20 sec at 95°, followed by 40 cycles of 1 sec at 95° and 20 sec at 60°; data capture was at end of extension period). Relative enrichment (for ChIP) or expression levels (for RNA, following cDNA synthesis) were obtained by determining the differences in Ct values compared with a reference; the values obtained this way were rescaled for presentation by addition of a constant. Primers are for coding regions or Abf1 binding sites in gene promoters and are listed in Table 2.

**Table 2:** Primers used in this study

Name	Sequence
YKT6-A	TATCCTGTCAGACCAGCATACACAC
YKT6-B	TGGAACTTGCCGTTAATGACTCCG
YKT6 1Fe	CCA AAA TTC GGC TCC TTT TCC CTT
YKT6 1Re	GAG CGA AAT ACA CCG ATG TAG TAG
VID27 1F E	CTA CAC CAT TGG TGA TTG GTG TTT
VID27 1R E	GAC TAC TTT GCT TTC AGT GCT GTC
TCM1 - 3	GACAGCTTCGACAACTTCACGCTT
TCM1-1	GCCTCCATCAGAGCTAGAGTTAAG
SPT15-A	CCC CTC TGA TAG CTG AGA TGT CGG GAT TCC
SPT15-C	CCAAGTTT CTCTTACGCGAGCTTTTTGG G
Rps28a-P1	CAAGCATCAAATCCCTTTTAAGCATATC
Rps28a-P2	CCATGATTGCTAGCTTGGTTTTCTGC
Rps28A-1	GGATAACAAAACCCCAGTCACTTTAGCC
Rps28a-2	GAC GAG CTT CAC GTT CAG ATT CCA TTA G
SNR6 1Fcr	TTC GCG AAG TAA CCC TTC GTG GA
SNR6 1Rcr	GTA AAA CGG TTC ATC CTT ATG CAG
RPN8 1Fe	TAC GGT AAG TAG TGA AGA CTC AC
RPN8 1Re	CAC TAT AGA TTG CTT AGC TGT TGG
RPN8 1Fcr	CGT TGG TGT CAT CTT AGG TGA TGC
RPN8 1Rcr	GGA CCA CTA TGA TAC CAT CCA ATG
RPL3 1F E	CTC ACG CAC ACT GGA ATG AAT GGC
RPL3 1R E	ATA TCC AGG AAG CAC GAA AGA GAC
RAD23 1Fe	CTA GGC TCG GTT TTT TAG TGA CCT
RAD23 1Re	AAA TTT CAA TTT CGC CAC CGA GCC
QCR8 1F	GCT GAT GTC TTA ACT GCG TTC TTG
QCR8 1R	CCG TAA TTT CCG ATC ACG CAT TTC
PIK1 1F E	CAT CAT AAG GCC ATT GTC ACC TTC
PIK1- 1Fcr	TGA TTTCA ACT CTA GTG AAT TCA CCC
PIK1 1R E	GGT AGG GTT CTT TTG TTT CAG TGC
PIK1- 1Rcr	ATG GTG ACG AGG ACC TGT ACT AGT
IPP1 1F E	GTC ATC GCA GAC GCT AAG GTT GTT
IPP1 1Fcr	TAC ACTACC AGA CAA ATT GGT GCC
IPP1 1R E	TCC TGT TCT ATA GAC CTA AGG GAC
IPP1 1Rcr	CCT TGG TGA TTT CTA ACT TGG CGT
CNB1 1Fe	GGA TTT GAT CGC GAA GAC GCT ATG
CNB1 1Re	TAA GAT GAT ACC CGG CCT TCC ACG
Cnb1 1fcr	GCTGCTCCTTCCAAAATTGTGGAT
Cnb1 1rcr	AACCTCCATTATACGTCCAGCAAG
ChrV-down	CACCCCGAAGCTGCTTTCACAATAC
ChrV-up	GGCTGTCAGAATATGGGGCCGTAGTA
ARO3 1F	GAA GCA GCT GCG TAT CTT CTC AAA
ARO3 1R	ATG CAG CAA GCA TAC TTT CCG ATG

### Computational analysis

Average profiles for memory effect and transcriptionally responsive genes were generated using data for 69 transcriptionally responsive targets and 64 memory effect targets (Yarragudi *et al.* 2007) containing unique Abf1 sites. ChIP-seq data were analyzed with the ArchTEx program (Lai *et al.* 2012) and Z-scored to normalize for variance between experiments after calculating the log_2_ ratio. To obtain Z-scores, sequence tags were first extended to a total length of 120 bp to account for the gap between forward and reverse reads and the total number of tags were summed at each base pair in the genome. Counts were divided at each base pair by the genomic average (120 bp × total sequence tags / genome size) and log_2_ values were generated for each ratio. The mean and variance of log_2_ ratios were calculated across the genome and normalized to N(0,1) for every base pair to yield Z-scores. Peaks were identified using MACS (*P* < 10^−3^) (Zhang *et al.* 2008); peaks identified using this relatively low stringency still generally showed decreased magnitude in *abf1-101 ts* yeast.

Predicted and *in vitro* nucleosome occupancy profiles were generated using the algorithm and data from Kaplan *et al.* (Kaplan *et al.* 2009). dA/dT motifs that disfavor nucleosome occupancy were defined as any 7bp sequence that contains at least 6 As or 6 Ts. The frequency of such sequences was examined in 150-bp windows surrounding each Abf1 motif, and these frequencies were averaged over the responsive *vs.* the memory-effect genes (Figure 4C). This analysis was performed for *S. cerevisiae* as well as for four closely related species, using aligned promoter sequences defined previously (Cliften *et al.* 2003; Kellis *et al.* 2003). Average expression noise was calculated using data from Newmann *et al.* which was centered at zero (Newman *et al.* 2006), and error bars were defined by bootstrapping; genes having “open” (DPN, depleted proximal nucleosome) and “closed” (OPN, occupied promoter nucleosome) promoter nucleosome configurations are taken from Tsui *et al.* (2011).

### Accession number

ChIP-seq data have been deposited at ArrayExpress under accession number E-MTAB-3208.

## Results

### Transcription continues at a subset of Abf1-regulated genes in two *abf1 ts* yeast mutants

**Figure 1:**
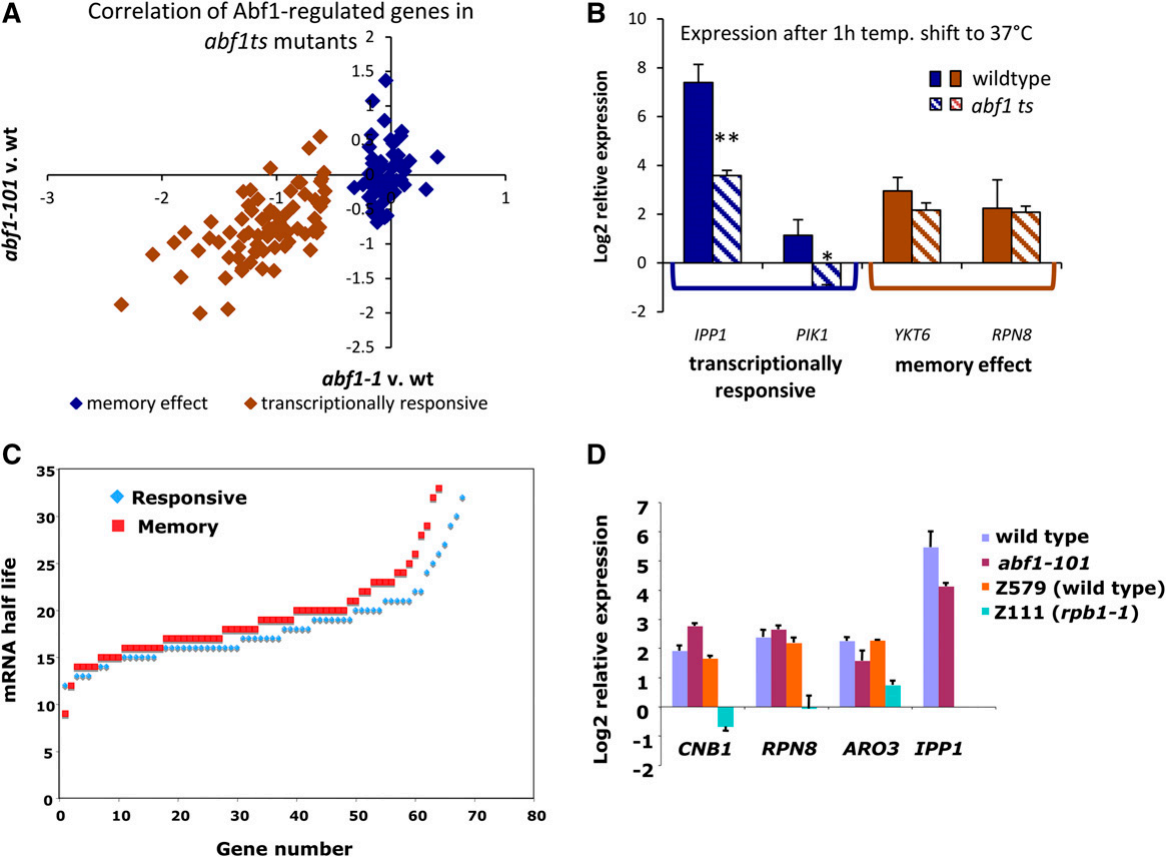
Expression of putatively Abf1-regulated genes in *abf1-101* and *abf1-1 ts* yeast at 37°. (A) Correlation of the change in expression for the two *abf1ts* mutants, *abf1-1* and *abf1-101*, compared with their respective wild-type strains after 1 hr at 37°. Microarray expression values are from (Yarragudi *et al.* 2007) (*abf1-1*) and (Badis *et al.* 2008) (*abf1-101*) for 133 genes designated as either memory effect (60, blue) or transcriptionally responsive (73, orange) in Yarragudi *et al.* (2007) (Badis *et al.* 2008; Yarragudi *et al.* 2007). R = 0.755, *P* = 3.0e-36. (B) Expression of transcriptionally responsive (*IPP1* and *PIK1*) and memory effect (*YKT6*, *RPN8*) Abf1 targets measured by cDNA/quantitative polymerase chain reaction analysis of transcript levels in wild type (BY4741) and *abf1-101 ts* yeast after 1 hr at 37°C. Expression is normalized to *SNR6*. Error bars represent the standard deviation for three biological replicates. *p-value of less than 0.05; **p-value of less than 0.01 (paired *t*-test). (C) mRNA half-lives from (Munchel *et al.* 2011) for 67 transcriptionally responsive genes and 64 memory effect genes. (D) Expression of two memory effect genes (*CNB1* and *RPN8*) in *abf1-101 ts* yeast, *rpb1-1 ts* yeast, and the corresponding wild type strains (BY4741 and Z579), after 1 hr at 37°C. Two transcriptionally responsive genes, *ARO3* and *IPP1*, are shown as positive controls for the temperature shift. Error bars represent the SD for 2−3 (two for *ARO3* only) biological replicates.

The categorization of putative Abf1 targets as “memory effect” or “responsive” genes was based on microarray analysis of gene expression using the *abf1-1 ts* mutant in a W303 background (Rhode *et al.* 1992; Yarragudi *et al.* 2007). Comparison of changes in gene expression from published microarray analysis of a distinct *ts* mutant, *abf1-101* (Loo *et al.* 1995), in a BY4741 background, revealed that this categorization is robust: changes in the two *ts* mutants strongly correlate (Figure 1A; R = 0.755; *P* = 2.977e-36) and the two classes of targets are for the most part well demarcated. Memory effect targets showed little change in expression in both the *abf1-1 ts* and *abf1-101 ts* strains, whereas the transcriptionally responsive targets showed on average a twofold decrease in expression in both backgrounds. We further validated the differing behavior of memory effect and transcriptionally responsive genes by comparing transcript levels for two genes belonging to each category in wild type and *abf1-101 ts* yeast after 1 hr at 37° (Figure 1B). The two transcriptionally responsive genes, *IPP1* and *PIK1*, which show decreased transcription in *abf1-1 ts* yeast (Yarragudi *et al.* 2007), display eight- and fourfold decreases in expression in *abf1-101 ts* yeast, respectively. In contrast, two genes identified as Abf1 “memory effect” genes in *abf1-1 ts* yeast, *YKT6* and *RPN8* (Yarragudi *et al.* 2007), show modest (less than twofold, *YKT6*) or no (*RPN8*) decrease in mRNA level in *abf1-101 ts* yeast (Figure 1A). These results indicate that an Abf1-mediated transcriptional memory effect can be observed in a distinct *ts* mutant background.

The distinct behavior of memory effect and transcriptionally responsive genes could be caused by differential transcript stability. To examine this possibility, we compared mRNA half-lives, as measured in a recent study in which a thiourea labeling protocol was used in a pulse-chase experiment (Munchel *et al.* 2011), for memory effect and transcriptionally responsive genes. As seen in Figure 1C, this feature does not distinguish these categories of transcript. Similar results were obtained using data from a more recent study that measured mRNA half-lives by a different method (Geisberg *et al.* 2014) (data not shown). As an independent test, we measured transcript levels for two memory effect genes in *abf1-101* and *rpb1-1 ts* yeast along with the corresponding wild type strains after 1 hr at 37° (Figure 1D). Transcription by RNA polymerase II ceases at 37° in the *rpb1-1* mutant, providing a measurement of mRNA stability (Nonet *et al.* 1987). Strains were grown and analyzed in parallel. Two “memory effect” genes, *CNB1* and *RPN8*, which show slightly increased transcript levels in *abf1 ts* yeast in both microarray experiments [log_2_ increase of 0.2 in both *abf1-1* and *abf1-101 ts* yeast (Badis *et al.* 2008; Yarragudi *et al.* 2007)] and by quantitative polymerase chain reaction (Figure 1D), exhibited more than fourfold decreased transcript levels in *rpb1-1 ts* yeast while showing no decrease in *abf1-101 ts* yeast (Figure 1D). A third putative “memory effect” gene, *RPL3* (also known as *TCM1*) behaved similarly; however, we show below that *RPL3* transcription does not depend on its Abf1 binding site. Transcript levels of *IPP1* decreased about threefold in the *abf1-101 ts* strain in this experiment, and *ARO3* transcript levels decreased approximately 1.6-fold. Although these decreases are somewhat less than seen in the experiment of Figure 1B, likely because of the variability in the temperature shift, they are consistent with the magnitude of the effects seen in microarray experiments and show that the temperature shift was effective (as do the decreased transcript levels seen in *rpb1-1* yeast). These results indicate that the continued expression of these Abf1 targets is not due to transcript stability.

### Memory effect genes are not distinguished by other contributing transcription factors or by lack of dependence on Abf1

Many yeast promoters bind multiple transcription factors. This could lead to redundancy in transcriptional activation, such that putative “memory effect” genes do not depend on Abf1 because of contributions from other activators. We compared transcription factor (TF) binding to memory effect and transcriptionally responsive genes using the CERES web tool (Morris *et al.* 2009) and found neither class to be enriched for binding sites for any specific TF (including Hsf1, which could conceivably be responsible for activating memory effect genes after the temperature shift to 37°). Furthermore, the average number of non-Abf1 TF binding sites within 500 bp of the starting ATG was very similar for the two classes, with memory effect genes averaging 1.5 TF binding sites/promoter and transcriptionally responsive genes averaging 1.24 TF binding sites/promoter. It therefore seems unlikely that other TFs make an important contribution to the distinction between memory effect and transcriptionally responsive promoters.

**Figure 2:**
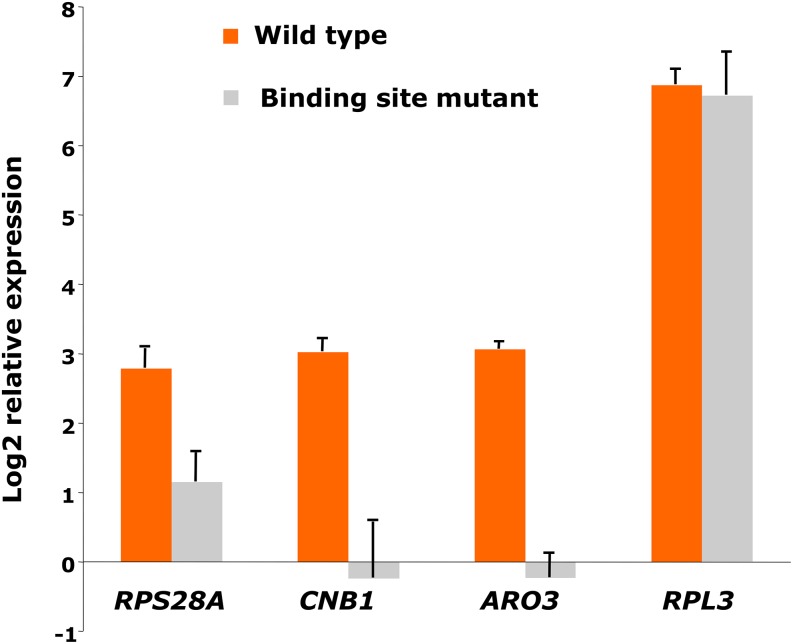
Dependence on Abf1 binding site for four putative Abf1-regulated genes. Expression analysis by cDNA/quantitative polymerase chain reaction of wild-type yeast and strains with mutated Abf1 binding sites in the promoter region of memory effect targets *RPS28A*, *CNB1*, and *RPL3*, and the transcriptionally responsive gene *ARO3*. Yeast were grown at 37° for 1 hr except for the experiment involving *RPS28A*, for which cells were grown at 30°. Expression is normalized to *SNR6*. Error bars represent the SD for 3−4 biological replicates.

A more direct test of Abf1 dependence would be to mutate promoter binding sites and examine the effect on transcript levels. Previous work has demonstrated the need for the Abf1 binding site at the promoter region of selected memory effect targets. Mutation of Abf1 binding sites at the *RPL3*, *QCR8*, *RPS28A*, *HIS7*, and *ADE5,7* promoters in plasmid-borne reporter constructs resulted in strongly decreased expression, whereas chromosomal mutations at the Abf1 binding site in the promoter of the memory effect gene *TOM6* resulted in a decrease in *TOM6* expression (Della Seta *et al.* 1990; Hornung *et al.* 2012; Lascaris *et al.* 2000; Yarragudi *et al.* 2004; Yarragudi *et al.* 2007). To test further the importance of the Abf1 binding site for transcription initiation at select memory effect genes, Abf1 binding sites were mutated in promoters of three memory effect targets, *RPL3*, *RPS28A*, and *CNB1*, and one transcriptionally responsive gene, *ARO3*, in their native chromosomal context by replacing the recognition sequence with a sequence to which Abf1 cannot bind. Expression analysis reveals that mutating the Abf1 binding site decreased transcription of *RPS28A* by about threefold, *CNB1* and *ARO3* by eightfold, and *RPL3* surprisingly not at all (Figure 2). These results indicate that although some “memory effect” genes may in fact not depend on Abf1 binding for their transcription, Abf1 binding is needed for full transcription initiation of at least a fraction of memory effect genes.

As an additional test for the functional importance of Abf1 sites in memory effect compared with transcriptionally responsive genes, we examined data on site conservation from previous work of Bulyk and colleagues (Mukherjee *et al.* 2004). Of 64 memory effect promoters having unique Abf1 binding sites, 41 showed conservation of the site in four related yeast species (*Saccharomyces mikatae*, *Saccharomyces kudriavzevii*, *Saccharomyces bayanus*, and *Saccharomyces paradoxus*) using a criterion of motif conservation within two standard deviations of the motif average, whereas 29 of 69 transcriptionally responsive promoters with unique Abf1 binding sites showed equivalent conservation. Abf1 sites in memory effect genes therefore do not appear to be less functionally important, based on evolutionary conservation, than those sites in transcriptionally responsive genes. Taken together, data from previous site mutation experiments and those shown here, together with information on evolutionary conservation, indicate that the memory effect is not likely explained by Abf1 being unimportant for transcriptional activity at these promoters.

### Abf1 dissociates from its binding site in *abf1ts* yeast

**Figure 3:**
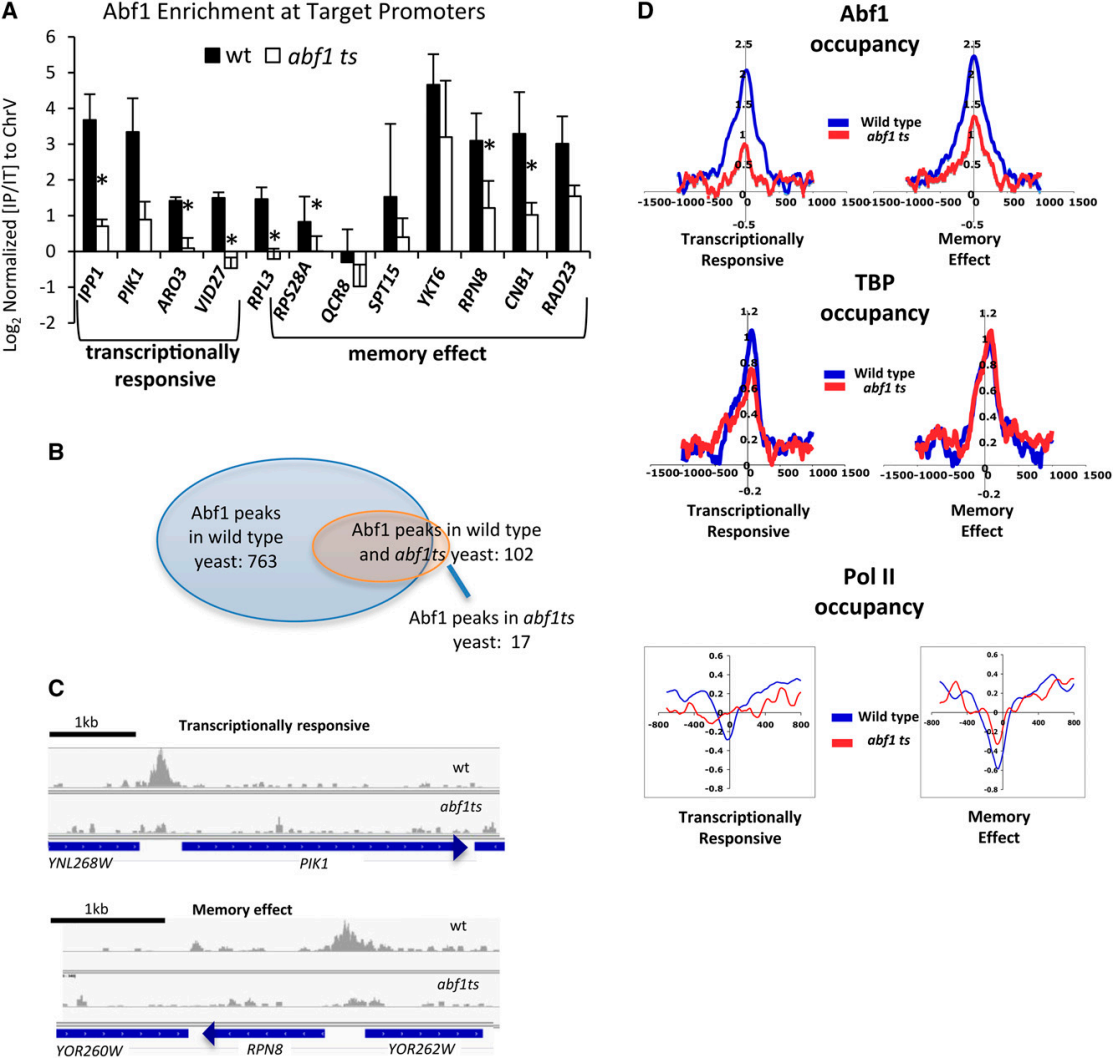
Effect on binding of Abf1 and the general transcription machinery of the *abf1-101 ts* mutation at the two classes of Abf1-regulated targets. (A) Abf1 enrichment was measured by ChIP followed by quantitative polymerase chain reaction at the indicated promoters, using primers spanning the Abf1 binding sites, in wild type and *abf1-101 ts* yeast. IP/input ratios were normalized to an open reading frame−free region of ChrV (Komarnitsky *et al.* 2000). Error bars represent the SD of three biological replicates. Note that, based on results of Figure 2, *RPL3* is categorized neither a transcriptionally responsive nor a memory effect gene. *p-value of less than 0.05. (B) Overlap of Abf1 enrichment peaks in wild-type yeast and *abf1-101 ts* yeast after 1 hr at 37°. (C) Screen shots of ChIP-Seq signals aligned to the budding yeast genome (saccer3, April 2011) for a transcriptionally responsive target, *PIK1* (*YNL267W*; upper panel), and a memory effect target *RPN8* (*YOR261C*; lower panel) captured in Integrative Genomics Viewer (Robinson *et al.* 2011). Scales are normalized to total reads per sample. (D) Averaged enrichment profiles for Abf1, TBP, and Rpb3 ChIP-Seq samples collected in wild-type and *abf1-101 ts* strains after 1 hr at 37°. Abf1 and TBP profiles represent average read density over 10-bp increments and are centered over the Abf1 binding site, whereas Pol II profiles are centered over transcription start sites and represent sliding windows averaged over 100 bp in increments of 10 bp. Log_2_ values of enrichment were averaged for 69 transcriptionally responsive targets and 64 memory effect targets containing a single Abf1 bindng site. Wild-type samples are in blue, *abf1 ts* samples in red. ChIP, chromatin immunoprecipitation; TBP, TATA-binding protein.

Another possible explanation for the distinct behavior of memory effect and transcriptionally responsive Abf1 targets is that loss of Abf1 binding occurs differentially in these two classes in *abf1 ts* yeast. Abf1 binding motifs in the promoters of these two classes were indistinguishable, making this seem less likely (Yarragudi *et al.* 2007). To further examine this possibility, individual loci were examined for Abf1 enrichment through ChIP in wild type and *abf1-101 ts* yeast. Abf1-regulated targets showed a clear decrease in binding of Abf1 in *abf1-101 ts* yeast at both transcriptionally responsive and memory effect genes after 1 hr at 37° (Figure 3A). There was a 4.5-fold average decrease in enrichment for four transcriptionally responsive targets and a 2.6-fold average decrease in Abf1 binding at seven memory effect targets. This difference in average fold decrease did not meet standard criteria for significance (*P* = 0.07), and the decrease in Abf1 enrichment at memory effect targets such as *RPN8* and *CNB1* confirms that transcript levels remain high in spite of Abf1 dissociating from its binding site at genes (at least some) that depend on their Abf1 binding site for normal levels of transcription (Figure 2). Nonetheless, these results left open the possibility that Abf1 may not be dissociating from memory effect targets as well as it does at transcriptionally responsive targets.

To expand this analysis, we used ChIP-Seq to examine Abf1 binding genome-wide in wild type and *abf1-101 ts* yeast after 1 hr at 37°. We identified a large number of Abf1 peaks in wild-type yeast, nearly all promoter-associated, in reasonable agreement with the number identified in a previous large-scale genome-wide association study (Harbison *et al.* 2004) and roughly comparable to the ~1200 identified in another recent study (Kasinathan *et al.* 2014) (Figure 3B). Only a fraction of the genes associated with these sites show decreased transcription in *abf1 ts* yeast (Badis *et al.* 2008; Yarragudi *et al.* 2007). Average Abf1 binding profiles were generated and centered over the Abf1 binding motif in the promoter regions of transcriptionally responsive and memory effect targets. Both classes of genes show a decrease in Abf1 enrichment levels when comparing the levels in wild-type yeast with those in *abf1-101 ts* yeast (Figure 3, C and D). The decrease was slightly greater at the transcriptionally responsive targets with log_2_ enrichment values centered on the Abf1 binding site decreasing from 2.0 in the wild type strain to 0.8 in the *abf1 ts* strain while enrichment at the memory effect targets decreased from 2.2 in the wild-type strain to 1.2 in *abf1-101 ts* yeast. The enrichment levels above background were essentially equivalent for both gene classes in wild type yeast, indicating that Abf1 binds equally well to both memory effect and transcriptionally responsive targets, consistent with the two classes being associated with indistinguishable motifs (Yarragudi *et al.* 2007). Thus, results of both ChIP-seq and conventional ChIP followed by quantitative polymerase chain reaction at select loci indicate substantial loss of Abf1 binding from both memory effect and transcriptionally responsive targets, with the loss being slightly greater from responsive gene promoters.

To determine whether association of the general transcription machinery differs at the two classes of Abf1 targets, enrichment of pre-initiation complex components, TATA-binding protein (TBP) and RNA Polymerase II (Rpb3 subunit), was examined by ChIP-Seq in wild type and *abf1ts* yeast after 1 hr at 37°. As expected, TBP association was observed at promoters of both memory effect and transcriptionally responsive promoters (Figure 3D). However, association of TBP at transcriptionally responsive targets decreased approximately 25% in the *abf1-101 ts* strain, whereas no decrease at all was observed at memory effect genes (Figure 3D). Similarly, Pol II enrichment downstream of Abf1 binding sites (*i.e.*, over coding regions) of Abf1-regulated targets decreased in *abf1-101 ts* yeast, while virtually no change was seen at memory effect genes (Figure 3D). These results are consistent with the distinct effects on mRNA expression levels for memory effect and transcriptionally responsive Abf1 targets being a direct result of altered response of the transcriptional machinery to decreased Abf1 binding at these two classes of genes.

### Memory effect and transcriptionally responsive promoters differ in nucleosome-forming propensity

**Figure 4:**
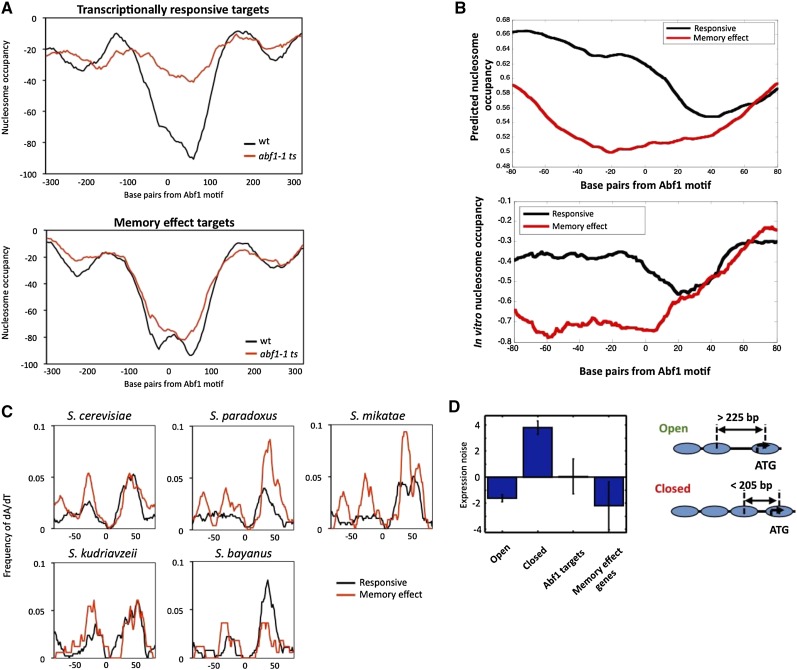
Transcriptionally responsive and memory effect targets differ in properties related to nucleosome occupancy. (A) *In vivo* average nucleosome occupancy in wild-type (WT, W303; green line) and *abf1-1ts* (red line) yeast after 1 hr at 37°C centered over the Abf1 binding site shows an open chromatin structure at memory effect targets in *abf1 ts* yeast. Top panel shows the average *in vivo* occupancy for 69 Abf1 transcriptionally responsive targets and bottom panel shows average occupancy for 64 memory effect targets (both limited to those containing only a single Abf1 binding site) as defined by Yarragudi *et al.* (2007). (B) (Kaplan *et al.* 2009); top panel) and *in vitro* (bottom panel) average nucleosome occupancy profiles, centered over the Abf1 binding site, for 69 transcriptionally responsive targets (black line) and 64 memory effect targets (red line). (C) Conservation of T-rich regions, based on frequency of 7-mers containing at least 6 A or T residues (see the section *Materials and Methods*), upstream of the Abf1 binding sites in memory effect promoters (blue) and transcriptionally responsive targets (black) in *S. cerevisiae* and four closely related yeast species. Averaged plots are centered over the Abf1 binding site. (D) Average gene expression noise (Newman *et al.* 2006) for genes having “Open” or “Closed” nucleosome occupancy configurations at their promoters (Tsui *et al.* 2011), and for transcriptionally responsive and memory effect genes as defined in the text.

Chromatin structure of promoter regions can affect transcriptional activity, as a more open chromatin structure can allow increased access to activators and general TFs, thus facilitating transcription (Zaret and Carroll 2011). To test whether chromatin structure contributes to the memory effect phenomenon, we used previously generated data to compare nucleosome occupancy for Abf1-regulated transcriptionally responsive and memory effect targets after 1 hr at 37° in wild-type and *abf1ts* yeast (Badis *et al.* 2008; Ganapathi *et al.* 2011). As expected, promoters for both classes of Abf1-regulated genes have an open chromatin structure over the Abf1 binding site in wild type yeast (Figure 4A) (Badis *et al.* 2008; Ganapathi *et al.* 2011; Hartley and Madhani 2009; Kaplan *et al.* 2009). This open chromatin structure was mostly lost in transcriptionally responsive gene promoters in *abf1-1 ts* yeast after 1 hr at 37°, indicating that dissociation of Abf1 from its binding site in these promoters is accompanied by generation of a closed chromatin conformation (Figure 4A). This finding is in accord with previous studies indicating that local domains of low nucleosome occupancy in the vicinity of Abf1 binding sites generally depend on Abf1 (Badis *et al.* 2008; Ganapathi *et al.* 2011). Remarkably, however, memory effect targets showed very little change in nucleosome occupancy over the Abf1 binding site in *abf1-1* yeast, instead exhibiting low nucleosome occupancy similar to that seen in wild-type yeast (Figure 4A). Essentially identical results were obtained in comparing nucleosome occupancy of transcriptionally responsive and memory effect genes in *abf1-101 ts* yeast (Supporting Information, Figure S1). These results suggest that open chromatin structure that persists in the promoter regions of the Abf1 memory effect genes after dissociation of Abf1 may allow the general transcription machinery continued access to the transcription start site, resulting in persistent expression.

We next asked what might cause the maintenance of low nucleosome occupancy observed at memory effect genes in *abf1 ts* yeast. One potential mechanism that could mark memory effect promoters would be recruitment of a particular accessory transcriptional activator, which might require Abf1 for its initial recruitment but not for continued binding and maintenance of an open chromatin structure. However, as mentioned previously, we observed no differential enrichment in type or number of TF binding sites between the promoters of memory effect and transcriptionally responsive Abf1 targets, including other general regulatory factors (Cbf1, Rap1, Reb1).

We then asked whether the propensity to form nucleosomes might differ in the vicinity of the Abf1 binding site of memory effect and transcriptionally responsive promoters; this could result in a greater tendency for one class than the other (presumably transcriptionally responsive over memory effect promoters) to be occupied by a nucleosome after loss of Abf1 binding. Previous work from the Widom and Segal labs measured nucleosome occupancy obtained upon packaging yeast genomic DNA into nucleosomes *in vitro*, and then developed an algorithm based on the results to predict relative nucleosome-forming propensity based on DNA sequence (Kaplan *et al.* 2009). We examined the *in vitro* nucleosome occupancy and predicted occupancy for memory effect and transcriptionally responsive promoter sequences, and found that memory effect promoter sequences showed considerably lower values in the vicinity of Abf1 binding sites (Figure 4B). Memory effect promoters showed enrichment for A/T-rich elements (see the section *Materials and Methods*), which are relatively unfavorable for nucleosome formation and can affect transcription of the associated gene, and which also have been shown to cooperate with Abf1 in transcriptional activation (Figure S2) (Lascaris *et al.* 2000; Segal and Widom 2009). This effect is confined to the upstream-flanking region of the motif and is almost completely absent in the downstream flanking region. The enrichment of A/T-rich elements at the upstream region of memory effect promoters is mostly for T-tracts and less evident for A-tracts (reading 5′ to 3′ on the upper strand), which is consistent with the observation that nucleosome-depleted regions are typically defined by T-tracts followed by A-tracts (Figure S2). These results strongly suggest that the differential response of memory effect and transcriptionally responsive promoters to loss of Abf1 binding is due at least in part to memory effect promoters showing a reduced propensity, based on DNA sequence, to form a closed chromatin structure upon loss of Abf1 binding in *abf1 ts* yeast.

The experimental conditions that reveal distinct behavior of memory effect and transcriptionally responsive Abf1 target genes are nonphysiological, in that they are observed using *abf1 ts* yeast. Therefore, to determine whether the observed differences in promoter type are physiologically significant, we examined DNA sequences of promoters of genes homologous to these two classes for nucleosome-forming propensity in four additional yeast species, *S. paradoxus*, *S. mikatae*, *S. kudriavzevii*, and *S. bayanus*. Similar to the results observed in *S. cerevisiae*, three of the four yeast species have increased levels of dA/dT upstream of the Abf1 binding site in the promoters of memory effect genes as compared to the promoters of transcriptionally responsive genes (Figure 4C). This conservation of the dA/dT tracts, and consequently the predicted lower nucleosome occupancy, suggests that this memory effect reflects an underlying and physiologically relevant property. Previous investigation of gene properties related to nucleosome occupancy has revealed two broad categories of yeast promoters: those having depleted proximal nucleosome, or more open, structure, and those having an occupied proximal nucleosome, or more closed, structure (Figure 4D) (Tirosh and Barkai 2008; Tirosh *et al.* 2009a). Promoters belonging to these two classifications differ on average in a number of properties, including TATA element, expression divergence, and expression noise (Tirosh and Barkai 2008; Tirosh *et al.* 2009a). Transcriptionally responsive and memory effect promoters are similar in their possession of consensus TATA elements and corresponding dependence on SAGA and TFIID (Huisinga and Pugh 2004). However, examination of the transcriptional noise associated with these two categories revealed a substantial difference. Memory effect promoters are associated with extremely low transcriptional noise, even lower than the average for promoters having depleted promoter nucleosome structure, while transcriptionally responsive promoters, although tending to have a nucleosome depleted region, have average noise (*i.e.*, similar to the genome-wide average) which is higher than other depleted proximal nucleosome promoters (Figure 4D). Thus, the memory effect that is characterized by continued transcription at Abf1-dependent promoters upon loss of Abf1 appears to reflect an evolved property that results in lower “on-off” switching, or noise, thus ensuring robust continued transcription of this class of genes.

## Discussion

Previous work by us and others has indicated the existence of two classes of genes whose transcription is regulated by Abf1: memory effect genes and transcriptionally responsive targets (Schroeder and Weil 1998; Yarragudi *et al.* 2007). These two categories were first defined based on analysis of genome-wide expression and ChIP-chip results in *abf1-1 ts* yeast (Yarragudi *et al.* 2007), and actually represent the two ends of a continuum. Abf1 targets were defined as genes whose promoters bind Abf1 *in vivo*, based on ChIP-chip (Harbison *et al.* 2004), and have an Abf1 binding motif or bind Abf1 *in vitro* (Yarragudi *et al.* 2007). Transcriptionally responsive targets were then defined as those whose transcription decreased at least 1.5-fold after 1 hr at 37° in *abf1-1 ts* yeast, whereas memory effect genes were defined as having log_2_ of transcriptional change less than 0.2. This categorization omits a substantial number of Abf1 targets with intermediate response, but is useful for exploring the varied behavior of Abf1-controlled genes.

Here we show that this same categorization holds for a different *abf1 ts* mutant, *abf1-101*, thus demonstrating that the effect is robust and not an artifact of one mutant. We also address a potentially trivial explanation for the difference between transcriptionally responsive and memory effect genes: that the former depend on Abf1 binding sites for their transcription and the latter do not. Previous work had indicated, in the context of plasmid reporter genes, that loss of Abf1 binding sites at several memory effect genes resulted in strongly diminished transcription (Della Seta *et al.* 1990; Hornung *et al.* 2012; Lascaris *et al.* 2000; Yarragudi *et al.* 2004; Yarragudi *et al.* 2007). Here we tested the effect of mutation of Abf1 binding sites in the native chromosomal context of three memory effect genes and one transcriptionally responsive gene (Figure 2). Two of the three memory effect genes showed four- to eightfold reduction in transcription, as did the transcriptionally responsive gene, whereas one, *RPL3*, showed no effect. Another study found mutation of the Abf1 binding site in the *TOM6* promoter in the chromosomal context resulted in about a threefold reduction in transcription [Figure 2E of (Hornung *et al.* 2012)]. Furthermore, conservation across *Saccharomyces* species of Abf1 binding sites is actually somewhat stronger among memory effect than transcriptionally responsive genes. Thus, although some “memory effect” genes may in fact not depend on Abf1 at all, this seems likely to represent a minor fraction of this category.

Consistent with the differential transcription of memory effect and transcriptionally responsive targets, ChIP-seq results show that although Abf1 binding is decreased in both classes, they differ in retention of the general transcription machinery in *abf1 ts* yeast. Finally, we show that memory effect and transcriptionally responsive genes display a major difference in their promoter chromatin structure, with memory effect gene promoters retaining low nucleosome occupancy in *abf1 ts* yeast while Abf1 binding sites of transcriptionally responsive promoters become occupied by nucleosomes. Importantly, transcriptionally responsive and memory effect genes defined in *abf1-1 ts* yeast show this same difference in nucleosome occupancy properties assessed in both *abf1-1* and *abf1-101* yeast, showing that this functional distinction according to an independent criterion also holds for distinct *abf1 ts* mutations. This distinguishing behavior is reflected by differential, sequence-directed propensity for nucleosome occupancy between the two classes, which displays evolutionary conservation and may be important for governing differential expression noise between the two classes.

Our ChIP-seq results for Abf1 complement previous work identifying Abf1 binding sites (Badis *et al.* 2008; Ganapathi *et al.* 2011; Harbison *et al.* 2004; Kasinathan *et al.* 2014). Early ChIP-chip studies indicated Abf1 binding to approximately 200 targets although expression data suggested there were additional loci affected by the loss of Abf1 (Harbison *et al.* 2004). A more recent study from our lab revealed that Abf1 contributes to low nucleosome occupancy at many of these additional loci (Ganapathi *et al.* 2011), while recent work from the Henikoff lab used a modified ChIP-seq protocol to identify 1258 binding sites for Abf1 (Kasinathan *et al.* 2014).

Although our ChIP-seq results showed that Abf1 dissociates from both classes of targets in *abf1 ts* yeast, transcriptionally responsive promoters showed a slightly more efficient loss of Abf1 than did memory effect genes. We believe this is more likely to reflect underlying, chromatin-mediated differences in these two classes of promoters than it is to be the cause of the difference. First, the Abf1 motif identified using MEME is identical for memory effect and transcriptionally responsive promoters (Bailey and Elkan 1994; Yarragudi *et al.* 2007). Second, although the average Abf1 occupancy at memory effect promoters is slightly higher than at transcriptionally responsive genes in *abf1 ts* yeast (Figure 3D), there is substantial overlap in the range of occupancies observed at the two promoter types. Rather, we suggest that the slightly more efficient eviction of Abf1 at the promoter region of the transcriptionally responsive targets in the *ts* mutant could be due to differential competition with the histone proteins. Upon shift to the restrictive temperature, the weak binding of the *abf1 ts* protein may not be strong enough to retain Abf1 at its binding site at transcriptionally responsive promoters because of their stronger propensity to assemble into nucleosomes, whereas the decreased likelihood of the nucleosome assembly due to nucleosome-disfavoring tracts (principally dT tracts) at the promoters of Abf1 memory effect targets could contribute to modest retention of Abf1 in the *ts* mutant at these loci. With weaker competition between Abf1 and the histone proteins, Abf1 is not evicted as efficiently.

The details of how the chromatin-mediated mechanism that we have uncovered here contributes to the memory effect seen at select Abf1-dependent genes remain unclear. It is difficult to ascertain the temporal limits of the memory effect, as yeast cells deficient in Abf1 function cannot progress through G1 into S phase (Rhode *et al.* 1992). The precise nature of the sequences that distinguish transcriptionally responsive and memory effect genes is not clear at present. Our initial efforts to interconvert responsive and memory effect Abf1 target genes by swapping sequences upstream of the Abf1 binding site did not succeed in clearly changing transcriptional response to loss of Abf1 binding in *abf1 ts* yeast. Thus, although sequence-directed differences in propensity for nucleosome occupancy appear important for distinguishing transcriptionally responsive from memory effect genes, other factors, including sequences downstream of Abf1 binding sites, may also contribute to this effect. An alternative and likely more efficient means to dissecting the sequences that distinguish transcriptionally responsive and memory effect promoters would be to measure this effect using engineered *abf1 ts* mutant strains of *Saccharomyces* species closely related to *S. cerevisiae*, as this would simultaneously monitor the effect of sequence changes in hundreds of promoters (Tirosh *et al.* 2009b). Future studies should lead to a more detailed understanding of the transcriptional memory effect studied here, and will likely provide insights into transcriptional responsiveness and the mechanisms underlying gene expression noise as well.

## References

[bib1] Ansari S. A. Ganapathi M. Benschop J. J. Holstege F. C. Wade J. T., 2012 Distinct role of Mediator tail module in regulation of SAGA-dependent, TATA-containing genes in yeast. EMBO J. 31: 44–57. 21971086 10.1038/emboj.2011.362 PMC3252575

[bib2] Badis G. Chan E. T. van Bakel H. Pena-Castillo L. Tillo D., 2008 A library of yeast transcription factor motifs reveals a widespread function for Rsc3 in targeting nucleosome exclusion at promoters. Mol. Cell 32: 878–887. 19111667 10.1016/j.molcel.2008.11.020 PMC2743730

[bib3] Bailey T. L. Elkan C., 1994 Fitting a mixture model by expectation maximization to discover motifs in biopolymers. Proc. Int. Conf. Intell. Syst. Mol. Biol. 2: 28–36. 7584402

[bib4] Cliften P. Sudarsanam P. Desikan A. Fulton L. Fulton B., 2003 Finding functional features in *Saccharomyces* genomes by phylogenetic footprinting. Science 301: 71–76. 12775844 10.1126/science.1084337

[bib5] de Boer M. Nielsen P. S. Bebelman J. P. Heerikhuizen H. Andersen H. A., 2000 Stp1p, Stp2p and Abf1p are involved in regulation of expression of the amino acid transporter gene BAP3 of *Saccharomyces cerevisiae*. Nucleic Acids Res. 28: 974–981. 10648791 10.1093/nar/28.4.974 PMC102570

[bib6] de Winde J. H. Grivell L. A., 1992 Global regulation of mitochondrial biogenesis in *Saccharomyces cerevisiae*: ABF1 and CPF1 play opposite roles in regulating expression of the QCR8 gene, which encodes subunit VIII of the mitochondrial ubiquinol-cytochrome c oxidoreductase. Mol. Cell. Biol. 12: 2872–2883. 1317009 10.1128/mcb.12.6.2872-2883.1992 PMC364482

[bib7] Della Seta F. Ciafre S. A. Marck C. Santoro B. Presutti C., 1990 The ABF1 factor is the transcriptional activator of the L2 ribosomal protein genes in *Saccharomyces cerevisiae*. Mol. Cell. Biol. 10: 2437–2441. 2183035 10.1128/mcb.10.5.2437-2441.1990 PMC360595

[bib8] Ganapathi M. Palumbo M. J. Ansari S. A. He Q. Tsui K., 2011 Extensive role of the general regulatory factors, Abf1 and Rap1, in determining genome-wide chromatin structure in budding yeast. Nucleic Acids Res. 39: 2032–2044. 21081559 10.1093/nar/gkq1161 PMC3064788

[bib9] Geisberg J. V. Moqtaderi Z. Fan X. Ozsolak F. Struhl K., 2014 Global analysis of mRNA isoform half-lives reveals stabilizing and destabilizing elements in yeast. Cell 156: 812–824. 24529382 10.1016/j.cell.2013.12.026 PMC3939777

[bib10] Harbison C. T. Gordon D. B. Lee T. I. Rinaldi N. J. Macisaac K. D., 2004 Transcriptional regulatory code of a eukaryotic genome. Nature 431: 99–104. 15343339 10.1038/nature02800 PMC3006441

[bib11] Hardy C. F. Sussel L. Shore D., 1992 A RAP1-interacting protein involved in transcriptional silencing and telomere length regulation. Genes Dev. 6: 801–814. 1577274 10.1101/gad.6.5.801

[bib12] Hartley P. D. Madhani H. D., 2009 Mechanisms that specify promoter nucleosome location and identity. Cell 137: 445–458. 19410542 10.1016/j.cell.2009.02.043 PMC2677553

[bib13] Ho S. N. Biggar S. R. Spencer D. M. Schreiber S. L. Crabtree G. R., 1996 Dimeric ligands define a role for transcriptional activation domains in reinitiation. Nature 382: 822–826. 8752278 10.1038/382822a0

[bib14] Hornung G. Oren M. Barkai N., 2012 Nucleosome organization affects the sensitivity of gene expression to promoter mutations. Mol. Cell 46: 362–368. 22464732 10.1016/j.molcel.2012.02.019 PMC3356688

[bib15] Huisinga K. L. Pugh B. F., 2004 A genome-wide housekeeping role for TFIID and a highly regulated stress-related role for SAGA in *Saccharomyces cerevisiae*. Mol. Cell 13: 573–585. 14992726 10.1016/s1097-2765(04)00087-5

[bib16] Kaplan N. Moore I. K. Fondufe-Mittendorf Y. Gossett A. J. Tillo D., 2009 The DNA-encoded nucleosome organization of a eukaryotic genome. Nature 458: 362–366. 19092803 10.1038/nature07667 PMC2658732

[bib17] Kasinathan S. Orsi G. A. Zentner G. E. Ahmad K. Henikoff S., 2014 High-resolution mapping of transcription factor binding sites on native chromatin. Nat. Methods 11: 203–209. 24336359 10.1038/nmeth.2766 PMC3929178

[bib18] Kellis M. Patterson N. Endrizzi M. Birren B. Lander E. S., 2003 Sequencing and comparison of yeast species to identify genes and regulatory elements. Nature 423: 241–254. 12748633 10.1038/nature01644

[bib19] Komarnitsky P. Cho E. J. Buratowski S., 2000 Different phosphorylated forms of RNA polymerase II and associated mRNA processing factors during transcription. Genes Dev. 14: 2452–2460. 11018013 10.1101/gad.824700 PMC316976

[bib20] Lai W. K. Bard J. E. Buck M. J., 2012 ArchTEx: accurate extraction and visualization of next-generation sequence data. Bioinformatics 28: 1021–1023. 22302569 10.1093/bioinformatics/bts063

[bib21] Lascaris R. F. Groot E. Hoen P. B. Mager W. H. Planta R. J., 2000 Different roles for abf1p and a T-rich promoter element in nucleosome organization of the yeast RPS28A gene. Nucleic Acids Res. 28: 1390–1396. 10684934 10.1093/nar/28.6.1390 PMC111049

[bib22] Loo S. Laurenson P. Foss M. Dillin A. Rine J., 1995 Roles of ABF1, NPL3, and YCL54 in silencing in *Saccharomyces cerevisiae*. Genetics 141: 889–902. 8582634 10.1093/genetics/141.3.889 PMC1206852

[bib23] Miyake T. Loch C. M. Li R., 2002 Identification of a multifunctional domain in autonomously replicating sequence-binding factor 1 required for transcriptional activation, DNA replication, and gene silencing. Mol. Cell. Biol. 22: 505–516. 11756546 10.1128/MCB.22.2.505-516.2002 PMC139751

[bib24] Miyake T. Reese J. Loch C. M. Auble D. T. Li R., 2004 Genome-wide analysis of ARS (autonomously replicating sequence) binding factor 1 (Abf1p)-mediated transcriptional regulation in *Saccharomyces cerevisiae*. J. Biol. Chem. 279: 34865–34872. 15192094 10.1074/jbc.M405156200

[bib25] Morris R. T. O’Connor T. R. Wyrick J. J., 2009 Ceres: software for the integrated analysis of transcription factor binding sites and nucleosome positions in *S. cerevisiae*. Bioinformatics 26: 168–174. 19959498 10.1093/bioinformatics/btp657

[bib26] Mukherjee S. Berger M. F. Jona G. Wang X. S. Muzzey D., 2004 Rapid analysis of the DNA-binding specificities of transcription factors with DNA microarrays. Nat. Genet. 36: 1331–1339. 15543148 10.1038/ng1473 PMC2692596

[bib27] Munchel S. E. Shultzaberger R. K. Takizawa N. Weis K., 2011 Dynamic profiling of mRNA turnover reveals gene-specific and system-wide regulation of mRNA decay. Mol. Biol. Cell 22: 2787–2795. 21680716 10.1091/mbc.E11-01-0028 PMC3145553

[bib28] Newman J. R. Ghaemmaghami S. Ihmels J. Breslow D. K. Noble M., 2006 Single-cell proteomic analysis of *S. cerevisiae* reveals the architecture of biological noise. Nature 441: 840–846. 16699522 10.1038/nature04785

[bib29] Nonet M. Scafe C. Sexton J. Young R., 1987 Eucaryotic RNA polymerase conditional mutant that rapidly ceases mRNA synthesis. Mol. Cell. Biol. 7: 1602–1611. 3299050 10.1128/mcb.7.5.1602-1611.1987 PMC365259

[bib30] Paul E. Zhu Z. I. Landsman D. Morse R. H., 2015 Genome-wide association of mediator and RNA polymerase II in wild-type and mediator mutant yeast. Mol. Cell. Biol. 35: 331–342. 25368384 10.1128/MCB.00991-14 PMC4295389

[bib31] Rhode P. R. Elsasser S. Campbell J. L., 1992 Role of multifunctional autonomously replicating sequence binding factor 1 in the initiation of DNA replication and transcriptional control in *Saccharomyces cerevisiae*. Mol. Cell. Biol. 12: 1064–1077. 1545789 10.1128/mcb.12.3.1064-1077.1992 PMC369538

[bib32] Robinson J. T. Thorvaldsdottir H. Winckler W. Guttman M. Lander E. S., 2011 Integrative genomics viewer. Nat. Biotechnol. 29: 24–26. 21221095 10.1038/nbt.1754 PMC3346182

[bib33] Schlecht U. Erb I. Demougin P. Robine N. Borde V., 2008 Genome-wide expression profiling, in vivo DNA binding analysis, and probabilistic motif prediction reveal novel Abf1 target genes during fermentation, respiration, and sporulation in yeast. Mol. Biol. Cell 19: 2193–2207. 18305101 10.1091/mbc.E07-12-1242 PMC2366881

[bib34] Schmitt M. E. Brown T. A. Trumpower B. L., 1990 A rapid and simple method for preparation of RNA from *Saccharomyces cerevisiae*. Nucleic Acids Res. 18: 3091–3092. 2190191 10.1093/nar/18.10.3091 PMC330876

[bib35] Schroeder S. C. Weil P. A., 1998 Genetic tests of the role of Abf1p in driving transcription of the yeast TATA box bindng protein-encoding gene, SPT15. J. Biol. Chem. 273: 19884–19891. 9677425 10.1074/jbc.273.31.19884

[bib36] Segal E. Widom J., 2009 Poly(dA:dT) tracts: major determinants of nucleosome organization. Curr. Opin. Struct. Biol. 19: 65–71. 19208466 10.1016/j.sbi.2009.01.004 PMC2673466

[bib37] Silve S. Rhode P. R. Coll B. Campbell J. Poyton R. O., 1992 ABF1 is a phosphoprotein and plays a role in carbon source control of COX6 transcription in *Saccharomyces cerevisiae*. Mol. Cell. Biol. 12: 4197–4208. 1324416 10.1128/mcb.12.9.4197-4208.1992 PMC360325

[bib38] Storici F. Resnick M. A., 2006 The delitto perfetto approach to in vivo site-directed mutagenesis and chromosome rearrangements with synthetic oligonucleotides in yeast. Methods Enzymol. 409: 329–345. 16793410 10.1016/S0076-6879(05)09019-1

[bib39] Thompson C. M. Young R. A., 1995 General requirement for RNA polymerase II holoenzymes in vivo. Proc. Natl. Acad. Sci. USA 92: 4587–4590. 7753848 10.1073/pnas.92.10.4587 PMC41989

[bib40] Tirosh I. Barkai N., 2008 Two strategies for gene regulation by promoter nucleosomes. Genome Res. 18: 1084–1091. 18448704 10.1101/gr.076059.108 PMC2493397

[bib41] Tirosh I. Barkai N. Verstrepen K. J., 2009 a Promoter architecture and the evolvability of gene expression. J. Biol. 8: 95. 20017897 10.1186/jbiol204 PMC2804285

[bib42] Tirosh I. Reikhav S. Levy A. A. Barkai N., 2009 b A yeast hybrid provides insight into the evolution of gene expression regulation. Science 324: 659–662. 19407207 10.1126/science.1169766

[bib43] Tsui K. Dubuis S. Gebbia M. Morse R. H. Barkai N., 2011 Evolution of nucleosome occupancy: conservation of global properties and divergence of gene-specific patterns. Mol. Cell. Biol. 31: 4348–4355. 21896781 10.1128/MCB.05276-11 PMC3209338

[bib44] Venditti P. Costanzo G. Negri R. Camilloni G., 1994 ABFI contributes to the chromatin organization of *Saccharomyces cerevisiae* ARS1 B-domain. Biochim. Biophys. Acta 1219: 677–689. 7948025 10.1016/0167-4781(94)90227-5

[bib45] Wapinski I. Pfeffer A. Friedman N. Regev A., 2007 Automatic genome-wide reconstruction of phylogenetic gene trees. Bioinformatics 23: i549–i558. 17646342 10.1093/bioinformatics/btm193

[bib46] Yarragudi A. Miyake T. Li R. Morse R. H., 2004 Comparison of ABF1 and RAP1 in chromatin opening and transactivator potentiation in the budding yeast *Saccharomyces cerevisiae*. Mol. Cell. Biol. 24: 9152–9164. 15456886 10.1128/MCB.24.20.9152-9164.2004 PMC517901

[bib47] Yarragudi A. Parfrey L. W. Morse R. H., 2007 Genome-wide analysis of transcriptional dependence and probable target sites for Abf1 and Rap1 in *Saccharomyces cerevisiae*. Nucleic Acids Res. 35: 193–202. 17158163 10.1093/nar/gkl1059 PMC1802568

[bib48] Zaret K. S. Carroll J. S., 2011 Pioneer transcription factors: establishing competence for gene expression. Genes Dev. 25: 2227–2241. 22056668 10.1101/gad.176826.111 PMC3219227

[bib49] Zhang Y. Liu T. Meyer C. A. Eeckhoute J. Johnson D. S., 2008 Model-based analysis of ChIP-Seq (MACS). Genome Biol. 9: R137. 18798982 10.1186/gb-2008-9-9-r137 PMC2592715

